# Oral Antibiotic Prophylaxis Lowers Surgical Site Infection in Elective Colorectal Surgery: Results of a Pragmatic Cohort Study in Catalonia

**DOI:** 10.3390/jcm10235636

**Published:** 2021-11-29

**Authors:** Josep M. Badia, Miriam Flores-Yelamos, Ana Vázquez, Nares Arroyo-García, Mireia Puig-Asensio, David Parés, Miguel Pera, Joaquín López-Contreras, Enric Limón, Miquel Pujol, Members of the VINCat Colorectal Surveillance Team

**Affiliations:** 1 Department of Surgery, Hospital General Granollers, 08348 Granollers, Barcelona, Spain; mfloresy@fphag.org (M.F.-Y.); narroyo@fphag.org (N.A.-G.); 2 School of Medicine, Universitat Internacional de Catalunya, 08195 Sant Cugat del Vallès, Barcelona, Spain; 3 Servei d’Estadística Aplicada, Universitat Autònoma de Barcelona, 08193 Bellaterra, Barcelona, Spain; Ana.Vazquez@uab.cat; 4 Department of Infectious Diseases, Hospital Universitari de Bellvitge, Spanish Network for Research in Infectious Diseases (REIPI RD16/0016/0005), 08907 L’Hospitalet del Llobregat, Barcelona, Spain; mpuiga@bellvitgehospital.cat (M.P.-A.); mpujol@bellvitgehospital.cat (M.P.); 5 Department of Surgery, Hospital Universitari Germans Trias i Pujol, 08916 Badalona, Barcelona, Spain; dapares@gmail.com; 6 Department of Surgery, Hospital del Mar, 08003 Barcelona, Catalonia, Spain; mpera@parcdesalutmar.cat; 7 Infectious Disease Unit, Hospital de la Santa Creu i Sant Pau–Institut d’Investigació Biomèdica Sant Pau, 08041 Barcelona, Barcelona, Spain; jlcontreras@santpau.cat; 8 VINCat Program, 08028 Barcelona, Catalonia, Spain; elimon@iconcologia.net; 9 Universitat de Barcelona, 08007 Barcelona, Catalonia, Spain

**Keywords:** colorectal surgery, surgical site infection, surgical wound infection, oral antibiotic prophylaxis, mechanical bowel preparation, preventative measures

## Abstract

Background: The role of oral antibiotic prophylaxis (OAP) and mechanical bowel preparation (MBP) in the prevention of surgical site infection (SSI) after colorectal surgery is still controversial. The aim of this study was to analyze the effect of a bundle including both measures in a National Infection Surveillance Network in Catalonia. Methods: Pragmatic cohort study to assess the effect of OAP and MBP in reducing SSI rate in 65 hospitals, comparing baseline phase (BP: 2007–2015) with implementation phase (IP: 2016–2019). To compare the results, a logistic regression model was established. Results: Out of 34,421 colorectal operations, 5180 had SSIs (15.05%). Overall SSI rate decreased from 18.81% to 11.10% in BP and IP, respectively (OR 0.539, CI_95_ 0.507–0.573, *p* < 0.0001). Information about bundle implementation was complete in 61.7% of cases. In a univariate analysis, OAP and MBP were independent factors in decreasing overall SSI, with OR 0.555, CI_95_ 0.483–0.638, and OR 0.686, CI_95_ 0.589–0.798, respectively; and similarly, organ/space SSI (O/S-SSI) (OR 0.592, CI_95_ 0.494–0.710, and OR 0.771, CI_95_ 0.630–0.944, respectively). However, only OAP retained its protective effect at both levels at multivariate analyses. Conclusions: oral antibiotic prophylaxis decreased the rates of SSI and O/S-SSI in a large series of elective colorectal surgery.

## 1. Introduction

Surgical site infections (SSI) are among the most common healthcare-related infections [1,2], and the most frequent postoperative complication. Due to the clean-contaminated nature of the procedure, rates of SSI after colorectal surgery are the highest among abdominal elective procedures, exceeding 20% in incidence studies with 30-day postoperative follow-up [3,4,5,6].

SSIs are associated with increased morbidity and mortality, and place considerable financial strain on healthcare systems, with increased consumption of antibiotics and prolonged length of hospital stays [7,8]. Organ/space SSI (O/S-SSI) in colorectal surgery triples hospital stay and is associated with a 23% rate of readmissions, 60% of reoperations and 29% need for intensive care [8].

About half of SSIs are thought to be avoidable [9], and numerous measures have been proposed for their prevention. Among them, some are exclusive to colorectal surgery, namely mechanical bowel preparation (MBP) and oral antibiotic prophylaxis (OAP). Nevertheless, the beginning of the 21st century saw a decline in the use of mechanical and oral bowel preparation (MOABP) after several studies compared MBP against non-preparation [10,11], and demonstrated that omitting MBP did not increase complications in colon and rectal surgery. In the United States, several surveys have documented the progressive reduction of the MOABP rate from 88% in 1990 to 36% in 2010 [12,13,14,15]. In Spain, a 2018 survey showed that MBP was used 95% of the time in rectal surgery, 59% in left colectomy and 28% in surgery of the right colon [16].

Several meta-analyses have shown that isolated mechanical bowel preparation is not an effective measure in decreasing the rate of infection [17,18,19,20,21,22]. On the contrary, data on oral antibiotics combined with MBP generated by randomized studies [23,24,25,26], meta-analyses [27,28,29,30,31,32], and observational studies [4,33,34] suggest that the combination of OAP with MBP plays a crucial role in reducing the risk of superficial, deep and organ/space SSI, as well as suture dehiscence, postoperative ileus, readmissions and mortality, without being associated with an increased risk of C. difficile infection [35].

In summary, there is still much debate about whether antibiotics should be administered only systemically or through a combination of oral and intravenous therapies before colorectal surgery [36,37,38].

To further understand the impact of the inclusion of OAP and MBP in a bundle of care, we analyzed a large series of elective colorectal procedures collected by a national infection surveillance network from 2011 to 2019.

## 2. Materials and Methods

This is a pragmatic prospective cohort study from VINCat, a voluntary network of 65 hospitals in Catalonia, Spain, that collect data on elective colorectal surgery for the purpose of quality improvement. The VINCat database is a nationally validated, outcomes-based program which aims to measure and improve healthcare-associated infections. Inclusion criteria were elective colorectal procedures wound class 2 (clean-contaminated) and 3 (contaminated). Prospective surveillance was performed by the Infection Control Team of each hospital to ensure appropriate data collection. Active mandatory post-discharge surveillance was performed up to day 30 post surgery by a multimodal approach including electronic review of clinical records (primary and secondary care), checking readmissions, checking emergency visits, and reviewing microbiological and radiological data.

In January 2016, a SSI reduction bundle was recommended to all hospitals in the network. The measures of the bundle were systemic antibiotic prophylaxis, mechanical bowel preparation, oral antibiotic prophylaxis, laparoscopic surgery, maintenance of normothermia, and use of a double-ring plastic wound edge retractor (Table 1). The oral antibiotics and recommended doses were neomycin 1 g, combined with metronidazole 500 mg (in 3 doses on the eve of surgery, 3 hours after the end of MBP).

To assess the effect of OAP and MBP in reducing SSI rate, the study matches two sequential time phases: a baseline phase (BP), from 2011 to 2015, before the bundle was introduced; and the bundle implementation phase (IP), from 2016 to 2019, during which the bundle was rolled out and increasingly embraced. Compliance with individual bundle elements was calculated in the IP group.

The primary outcome variable was the development of a SSI within 30 days of operation. The definitions of the Centers for Disease Control and Prevention (CDC) were followed. SSIs were defined as superficial, deep and/or organ space (O/S-SSI) [39,40].

Secondary outcome measures were length of hospital stay (LOS) and mortality.

### Statistical Analysis

Data are summarized as frequencies and proportions for categorical variables. For continuous variables, mean and standard deviation are presented. Infection rates were expressed as cumulative incidence, that is, the crude percentage of operations resulting in SSI/number of surgical procedures.

Groups were compared using the Pearson chi-squared test or the Fisher exact test for categorical variables, and Student’s *t*-test or ANOVA test for continuous variables. To describe the relationship between two qualitative variables, contingency tables were used. To characterize the infection, a logistic regression model was performed.

The results of modelization are presented in terms of odds ratio (OR) or estimated infection rates, with the corresponding 95% confidence intervals (CI_95_). The significance level was set at 0.05 in all tests. The results were analyzed using software SAS v9.4 (SAS Institute Inc., Cary, NC, USA).

Data belong to a large non publicly available national database. As all eligible patients were included in the pragmatic design, informed consent was not obtained. The study was conducted as a performance improvement project and approved by the Ethics in Research Committee with code 2021006.

The project was registered with the ClinicalTrials.gov Identifier: NCT04496635 and was reported according to the “Strengthening the Reporting of Observational Studies in Epidemiology (STROBE)” statement [41].

## 3. Results

The demographic and surgical characteristics of the patients are shown in Table 2. During the 9 years of the study, 34,421 colorectal operations were analyzed, 17,643 included in the BP group and 16,778 in the IP group. There were statistical differences in some of the analyzed items between the groups, the most clinically relevant being an increase in the use of laparoscopy from 57.90% to 73.28% (*p* < 0.0001).

In all cases, information was obtained on the application of the bundle measures, although the level of adherence to each of the measures was variable, from 100% for systemic antibiotic prophylaxis or the use of laparoscopy, to 65% for application of OAP or MBP.

### 3.1. Outcomes Comparison BP–IP Groups

A total of 5180 patients suffered SSIs (15.05%). This rate decreased from 18.81% in BP to 11.10% in IP (OR 0.539, CI_95_ 0.507–0.573, *p* < 0.0001) after implementation of the bundle. O/S-SSI rate also declined during the studied period from 9.8% to 6.5% (OR 0.633 CI_95_ 0.584–0.687, *p* < 0.0001), (Table 3).

Mean LOS decreased from 11.8 days in BP to 9.4 days in IP (CI_95_ for the difference 1.917–2.8646, *p* < 0.0001). Administration of OAP had an independent effect on the decrease of LOS (10.5 vs. 8.2 days; CI_95_ 1.746–2.848, *p* < 0.0001). Likewise, use of MPB reduced LOS by 1.3 days (9.9 vs. 8.6 days; CI_95_ 0.673–1.909, *p* < 0.0001).

With regard to 30-day patient mortality, it decreased from 1.28% in BP to 0.79% in IP (X^2^ = 24.2518, *p* < 0.0001).

### 3.2. Overall SSI

Information on OAP was recorded in 9741 procedures, of which it was implemented in 7028 cases. SSI developed at any level in 573 patients who received OAP (8.15%) and 374 who did not (13.79%, Table 4). Hence, OAP was related to a significant decrease in SSI rate (X^2^ = 69.24, *p* < 0.0001). In the univariate analysis (Table 5), administration of OAP was an independent predictive factor associated with less SSI (OR 0.555, CI_95_ 0.483–0.638).

MBP was used in 7886 colorectal procedures, out of 9980 records. Patients who received MBP had an SSI rate of 8.9%, compared to 12.5% who did not (X^2^ = 23.86, *p* < 0.0001). Use of MBP was an independent predictive factor associated with less SSI (OR, 0.686, CI_95_ 0.589–0.798) (Table 5).

**Figure 1:**
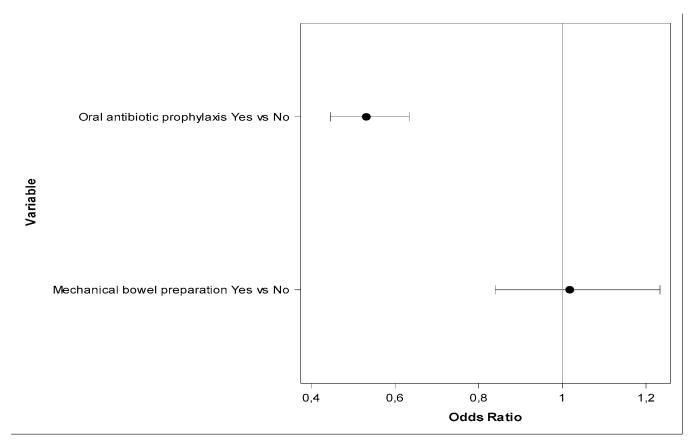
OR with 95% confidence interval for variables OAP and MBP from multivariate logistic model, with response variable SSI and antibiotic prophylaxis, laparoscopy, OAP, MBP and wound retractor as independent variables.

However, in a multivariate analysis of the six measures included in the bundle, OAP maintained its statistical significance (OR 0.531, CI_95_ 0.445–0.634; *p* < 0.0001), but MBP was not associated with a significant decrease in SSI rate (OR 1.017, CI_95_ 0.839–1.23; *p* = 0.8584) (Table 5, Figure 1). Adequate systemic antibiotic prophylaxis, laparoscopic technique, and use of double-ring wound retractor also showed a significant protective effect, whereas maintenance of normothermia did not.

Increasing bundle compliance was associated with lower SSI risk. Patients who received one measure of the bundle had a 16.7% of overall SSI rate, compared to those receiving all six measures, who had a rate of 5.8% (OR 3.250, CI_95_ 1.555–6.794; *p* < 0.0001).

The effects of OAP were further analyzed by year of surgery (Table 6), and their protective effects remained stable across 2016–2019.

### 3.3. Organ-Space SSI

Patients who received OAP had an O/S-SSI rate of 4.6%, compared to 7.5% who did not (X^2^ = 32.06, *p* < 0.0001, Table 3). Administration of OAP was associated with a lower rate of O/S-SSI (OR, 0.592, 0.494–0.710) (Table 7).

The group of patients with MBP had an O/S-SSI rate of 5.0%, compared to 6.4% who did not (X^2^ = 6.3486, *p* = 0.0117), with MBP an independent factor associated with lower O/S-SSI in univariate analyses (OR, 0.771, CI_95_ 0.630–0.944) (Table 7).

Nevertheless, the multivariate analyses showed that OAP, but not MBP, remained as a protective factor for O/S-SSI (OR 0.585, CI_95_ 0.465–0.735, *p* < 0.0001 (Table 7).

## 4. Discussion

In this study, based on data from the colectomy-targeted VINCat database, the implementation of a bundle of six measures including OAP and MBP was associated with a significant decrease in SSI, O/S-SSI, LOS and mortality. OAP was an independent protective factor against SSI and O/S-SSI, both in univariate and multivariate analyses. This effect remained stable during the study time period.

OAP and MBP are controversial SSI preventative measures which are exclusive for colorectal surgery [36,37,38]. For the last two decades, the development of multimodal rehabilitation programs in colorectal surgery [42], and the publication of several conflicting studies, have fueled this controversy, leading to a significant decrease in MBP and OAP prescription rates worldwide. In 2017, a European survey showed an oral prophylaxis use of only 11% and a routine use of MBP of 29.6% [43].

Nevertheless, in recent years, some authors have begun to advocate for a re-evaluation of the indication of OAP alone or combined with MBP in patients undergoing elective colon or rectal surgery [29,44,45,46].

In 2019, an innovative superiority trial compared MOABP with no bowel preparation in elective colectomy [47]. The analysis of 396 patients did not find differences in SSI rates (7% vs. 11%; OR 1.65, CI_95_ 0.80–3.40; *p* = 0.17). However, even the authors acknowledge the trial was underpowered to detect this small difference in SSI rate (4%) and to demonstrate the possibility of a benefit in using MOABP, when compared to no preparation [47,48].

Another multicenter randomized trial compared no MOABP with OAP administration (without MBP) in 565 patients. OAP was associated with a reduction in the risk of SSI, although this was only significant for superficial SSIs, due to the low number of infections detected in each subgroup [49].

While waiting for the confirmation of this new evidence, authorized surgical researchers think that the MOABP strategy should not be disregarded, but rather adjusted to the new findings in gut microbiome [45]. Non-antibiotic approaches to limit SSI-related pathogens while preserving beneficial bacteria of the intestinal microbiota have been proposed [45]. In this context of conflicting reports, the debate continues. In the meantime, it seems that the classic formulation of MOABP should be pragmatically tested, and cohort studies including a large number of procedures would probably be able to clarify the situation.

Pragmatic studies focus on an individual decision maker within an actual real-world situation, and put practical solutions above philosophical discussions. This approach to the problem within its broadest context seeks to better understand and ultimately solve the problem [50]. This present pragmatic prospective cohort study analyzed a large national database over nine years and was designed to answer the question whether OAP alone or in combination with MBP lowers SSI, among other postoperative complications.

In summary, the use of OAP correlates with lower SSI, OS-SSI, LOS, and mortality. We think our results can be generalized and are in accordance with those of recent randomized trials [51] and cohort studies [52,53], where MOABP was shown to be significantly associated with reduced risk of SSI in open and laparoscopic colorectal surgery.

The most recent guidelines of several scientific societies also recommend the inclusion of MOABP in their bundles of care for colorectal surgery, even in the setting of enhanced recovery after surgery (ERAS) programs [54,55].

### Strengths and Limitations of the Study

This work has several limitations. First, even though the sequential study groups are to some extent homogeneous, some other interventions implemented during the frame time of the study, such as the increased use of the laparoscopic technique, may have interfered with the results. Second, due to the characteristics of the data collection, some related secondary data, for example anastomotic leakage, were not analyzed. However, we think that the data are consistent, precise and reliable, if we give due consideration to the high number of procedures evaluated and its prospective origin, within the framework of a well-consolidated infection surveillance program.

## 5. Conclusions

A SSI reduction bundle including OAP and MBP was associated with a significantly reduced risk of overall SSI and O/S-SSI. OAP was a strong and independent protective factor against all types of SSI, including O/S-SSI. There is also evidence to suggest that the value of OAP did not decrease over time. Given the strength of the sample size, this may support the routine use of OAP combined with MBP in elective colorectal surgery.

Future prospective trials should clarify the role of OAP in the absence of MBP in this type of surgery.

## Figures and Tables

**Table 1:** Measures included in the VINCat colorectal bundle.

“Adequate” antibiotic prophylaxis	According to hospital protocol Start 30–60 min before incision. Redosification when indicated. Do not prolong > 24 h.
Mechanical bowel preparation	Day before of the procedure
Oral antibiotic prophylaxis	Day before of the procedure
Laparoscopic surgery	
Maintenance of normothermia	Goal: >36° at the end of operation
Double-ring plastic wound edge retractor	In open or laparoscopic surgery

**Table 2:** Colorectal surgery characteristics of the patients included in the study (2011–2019).

	Overall	BP (2011–2015)	IP (2016–2019)	*p*
Number of procedures	34,421	17,643	16,778	
Age, years (SD)	68.67 (12.40)	68.86 (12.37)	68.46 (12.43)	0.0026
Sex, male (%)	20,709 (60.16%)	10,703 (60.66%)	10,006 (59.64%)	0.0518
Adequate surgical prophylaxis (%) *	28,800 (83.79%)	15,069 (85.51%)	13,731 (81.98%)	<0.0001
Mean duration of intervention, minutes (SD)	177.50 (76.12)	175.35 (76.13)	179.75 (76.05)	<0.0001
ASA score > 1 (%)	32,427 (94.62%)	16,629 (94.39%)	15,798 (94.86%)	0.0548
Laparoscopy (%)	22,415 (65.39%)	10,180 (57.90%)	12,235 (73.28%)	<0.0001
NNISS ≥ 1 (%)	10,532 (30.60%)	6044 (34.26%)	4488 (30.60%)	<0.0001

BP: Baseline phase, IP: Implementation phase. NNISS: National nosocomial infections surveillance system. * Adequate surgical prophylaxis: type of antibiotic according to local guidelines, in addition to correct timing, dosage and duration.

**Table 3:** Colorectal surgery SSI rates during the study period (2011–2019) and comparison of IP and BP.

	Overall SSI (2011–2019)	BP (2011–2015)	IP (2016–2019)	OR (CI_95_)	*p*
SSI	15.05%	18.81%	11.10%	0.539 (0.507–0.573)	<0.0001
Superficial-SSI	5.3%	6.3%	3.6%	0.549 (0.496–0.608)	<0.0001
Deep-SSI	2.5%	3.4%	1.6%	0.448 (0.385–0.521)	<0.0001
O/S-SSI	8.2%	9.8%	6.5%	0.633 (0.584–0.687)	<0.0001

BP: Baseline phase, IP: Implementation phase. SSI: Surgical Site Infection. O/S-SSI: Organ-Space SSI.

**Table 4:** SSI rates according to the use of OAP and MBP.

	OAP		MBP	
	Yes	Not	Yes	Not
SSI	8.15%	13.79%	8.9%	12.5%
Superficial SSI	2.5%	4.39%	2.65%	4.73%
Deep SSI	1.04%	1.84%	1.23%	1.34%
Organ-space SSI	4.6%	7.5%	5.0%	6.4%

SSI: surgical site infection; OAP: oral antibiotic prophylaxis; MBP: mechanical bowel preparation.

**Table 5:** Effect of OAP and MBP on overall SSI.

	Univariate			Multivariate *		
	OR	CI_95_	*p*	OR	CI_95_	*p*
OAP	0.555	0.483–0.638	<0.0001	0.531	0.445–0.634	<0.0001
MBP	0.686	0.589–0.798	<0.0001	1.017	0.839–1.23	0.8584

SSI: surgical site infection; OAP: oral antibiotic prophylaxis; MBP: mechanical bowel preparation. * in the multivariate logistic model were included the six measures of the bundle (systemic antibiotic prophylaxis, laparoscopy, normothermia, OAP, MBP and wound retractor).

**Table 6:** Trends in the effect of OAP on overall SSI during the study period.

	Year of Intervention			
	2016	2017	2018	2019
Overall SSI with OAP	7.2%	8.7%	8.9%	7.3%
Overall SSI without OAP	13.0%	15.6%	13.9%	11.4%

SSI: surgical site infection; OAP: oral antibiotic prophylaxis.

**Table 7:** Effect of OAP and MBP on organ-space SSI.

	Univariate			Multivariate *		
	OR	CI_95_	*p*	OR	CI_95_	*p*
OAP	0.592	0.494–0.710	<0.0001	0.585	0.465–0.735	<0.0001
MBP	0.771	0.630–0.944	0.0117	1.101	0.854–1.418	0.4575

SSI: surgical site infection; OAP: oral antibiotic prophylaxis; MBP: mechanical bowel preparation. * in the multivariate logistic model were included the six measures of the bundle (systemic antibiotic prophylaxis, laparoscopy, normothermia, OAP, MBP and wound retractor).

## Data Availability

Restrictions apply to the availability of these data, which belong to a national database and are not publicly available. Data were obtained from VINCat and are only available with the permission of the VINCat Technical Committee.

## Appendix A. Members of the VINCat Colorectal Surveillance Team

Cecilia Diaz, Department of Anaesthesiology. Hospital Universitari Sant Pau. Barcelona, Spain. CDiez@santpau.cat; Marta Piriz Infection control team. Hospital Universitari Sant Pau. Barcelona, Spain. Mpirizm@santpau.cat; Domenico Fraccalvieri, Department of Surgery, Hospital Universitari de Bellvitge. L’Hospitalet de Llobregat, Spain. dofrac@yahoo.es; Anna Abad, Department of Anaesthesiology, Hospital Universitari Vall d’Hebrón, Barcelona, Spain. aat23865@yahoo.es; Lucrecia López, Infection control team. Hospital de Sant Joan Despí Moisès Broggi, Spain. lucre.lopez@sanitatintegral.org.
